# Supplementary material for: Reduction in organ–organ friction is critical for corolla elongation in morning glory
Source: Commun Biol. 2021 Mar 5;4:285. doi: 10.1038/s42003-021-01814-x (PMC7935917; doi:10.1038/s42003-021-01814-x)
Supplement: Supplementary file 2 — Supplementary Information [file 42003_2021_1814_MOESM2_ESM.pdf]

Supplementary Information for

Reduction in organ–organ friction is critical for corolla elongation in morning glory

Authors: Ayaka Shimoki, Satoru Tsugawa, Keiichiro Ohashi, Masahito Toda, Akiteru Maeno, Tomoaki Sakamoto, Seisuke Kimura, Takashi Nobusawa, Mika Nagao, Eiji Nitasaka, Taku Demura, Kiyotaka Okada, Seiji Takeda

Supplementary Figures 1~8

Supplementary Tables 1 and 2

Supplementary Data Files 1 and 2

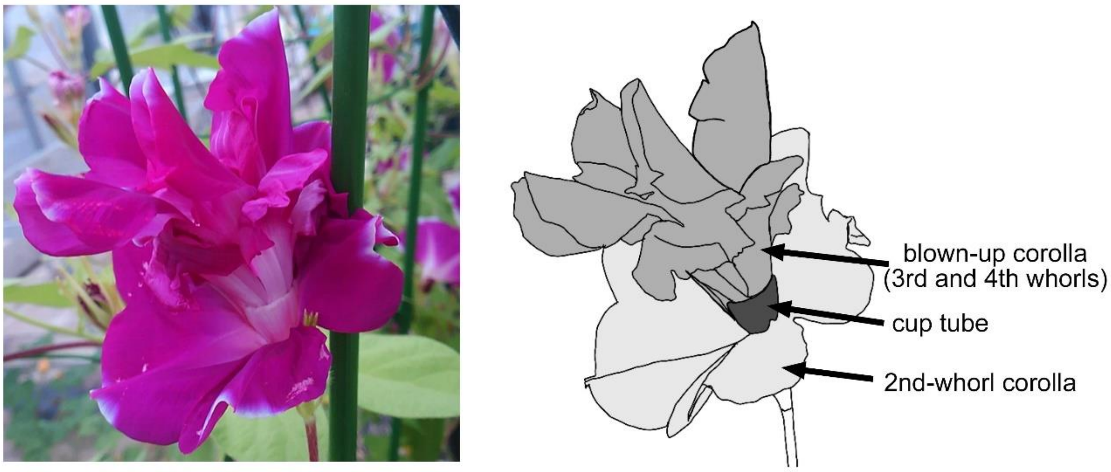

Supplementary Fig. 1. The combination of a cup tube with a homeotic mutation results in a blown-up corolla at the center of the flower, which has high ornamental value. Line ID; Q1096. The left panel shows an image of a flower, and the right panel shows a cartoon explaining each part of a flower.

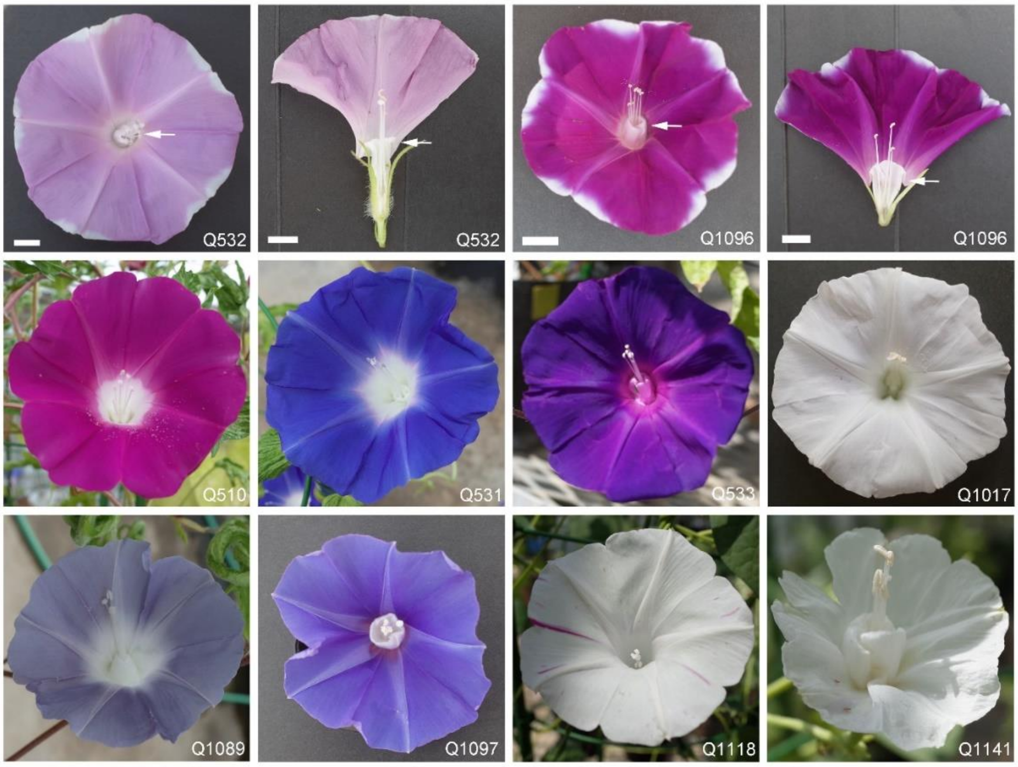

Supplementary Fig. 2. Cup flower lines used in this study. The line ID is indicated in each panel. The arrows in Q532 and Q1096 show the folded tubes. Q1118 is a wild-type *I. purpurea* line, from which the cup flower line Q1141 segregated. Scale bars; 1 cm.

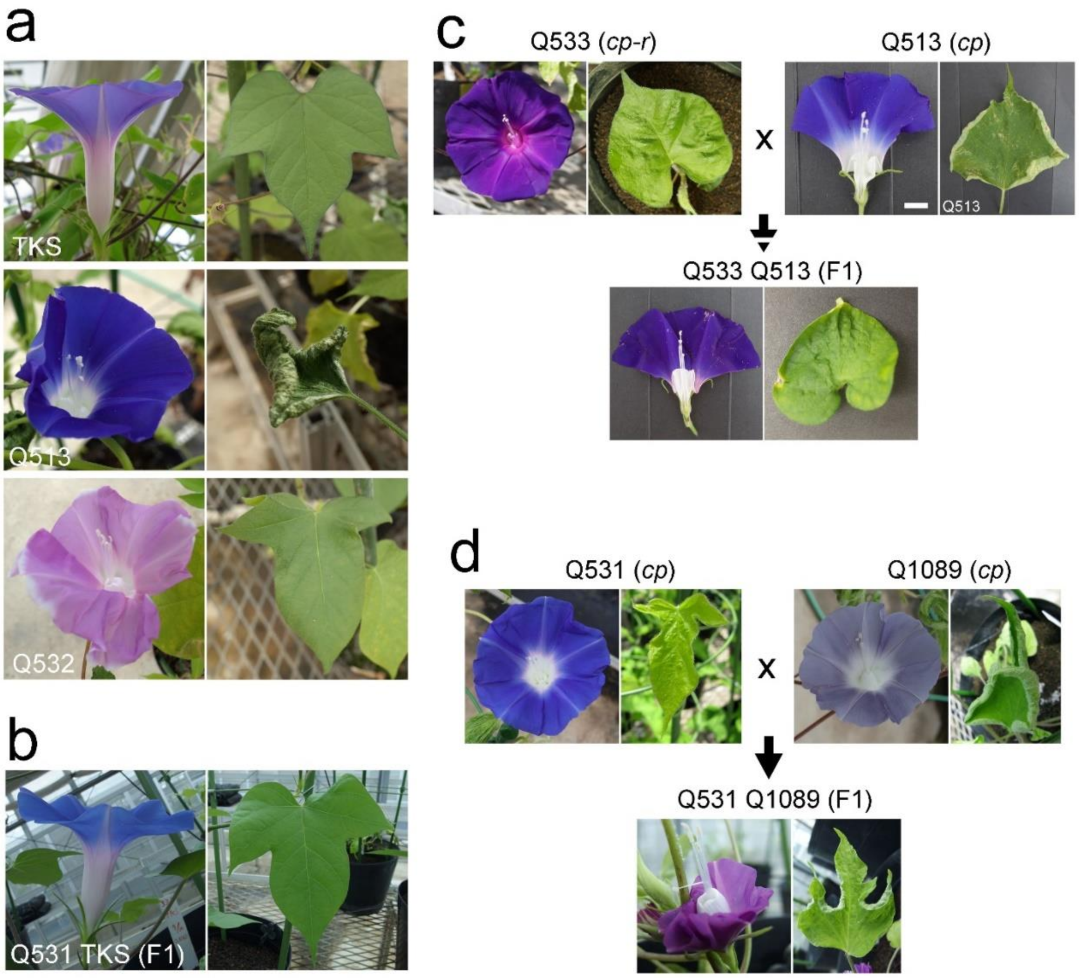

Supplementary Fig. 3. Leaf morphology and allelism test for the cup flower locus. **a,** Flowers and leaves of TKS, Q513 (*cp*) and Q532 (*cp-r*). Note that the Q532 *cp-r* line bears normal leaves. **b,** A cross between wild-type TKS and *cp* resulted in normal flowers in the F1 generation. **c and d,** Crosses between *cp-r* and *cp* (c), and *cp* and *cp* (d); both produced cup-shaped flowers in F1 generation.

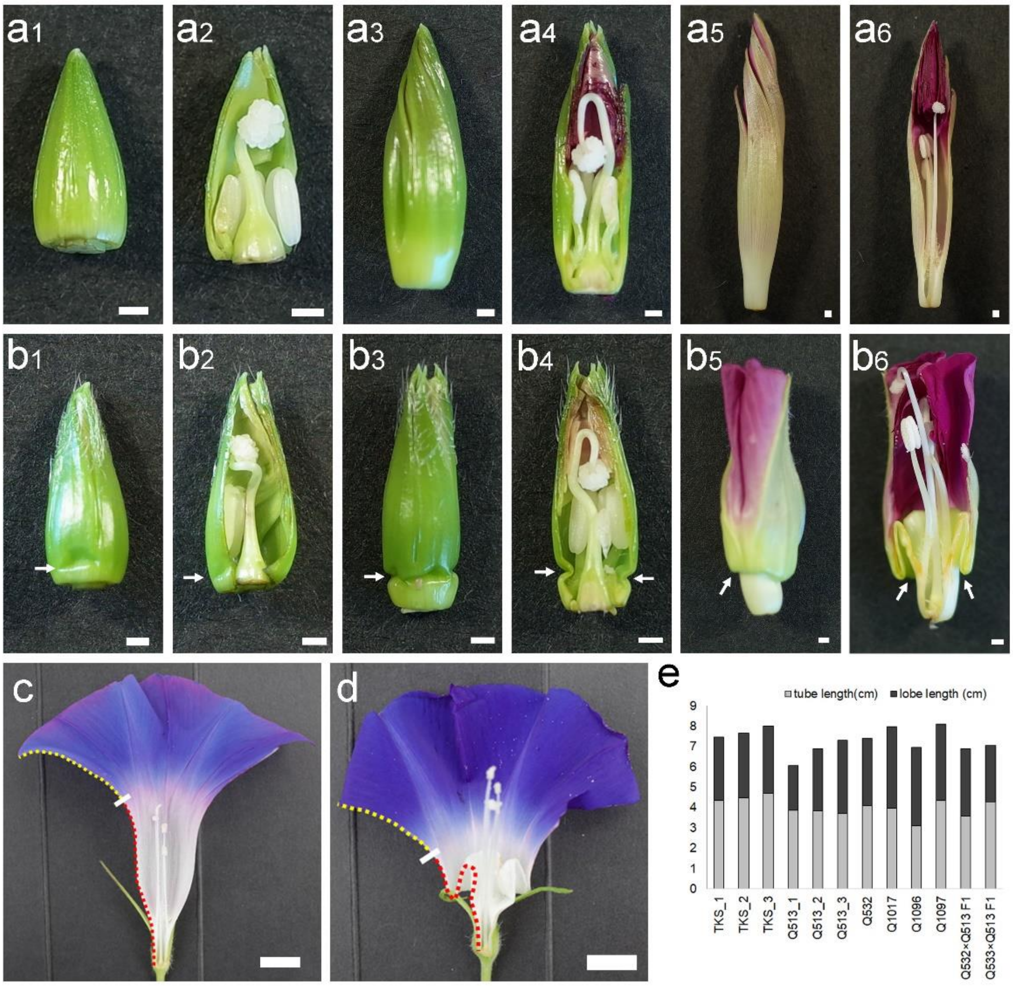

Supplementary Fig. 4. Developing floral buds. **a,** TKS. **b,** Q513. Sepals were removed in all the floral buds. The panels with small numbers (1, 3, and 5) and panels 2, 4, and 6 show intact and half-cut buds, respectively. The arrows in (b) indicate the cavity and deep folding. Scale bars: 1 mm. **c and d,** Half-cut flowers for measuring the length of the tube (red broken line) and lobe (yellow broken line) parts. Scale bars: 1 cm. **e,** Length of tube and lobe parts in TKS and cup flower mutants. Note that GSTs on TKS have fully developed at early stage (a_1) that corresponds to the stage of corolla folding in cup flower mutants (b_1) (see also Fig. 3).

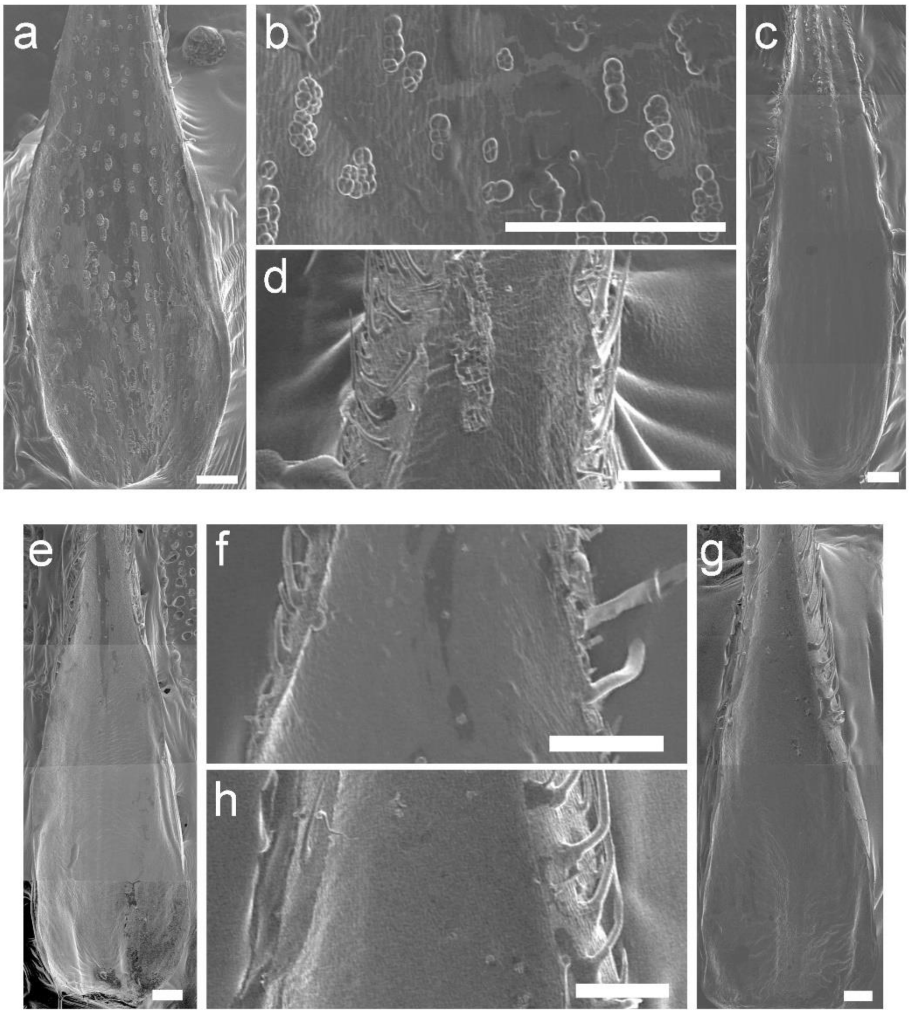

Supplementary Fig. 5. Glandular secretory trichomes (GSTs) on sepals. **a and b,** TKS. A cluster of GSTs has formed on the adaxial side of the sepals. **c and d,** Adaxial side of sepals in Q513, showing almost no GSTs in the basal and middle regions and few GSTs in the apical region. **e–h,** Adaxial side of sepals of Q532 (e and f) and Q1096 (g and h), presenting fewer and smaller GSTs. Scale bars: 500 µm.

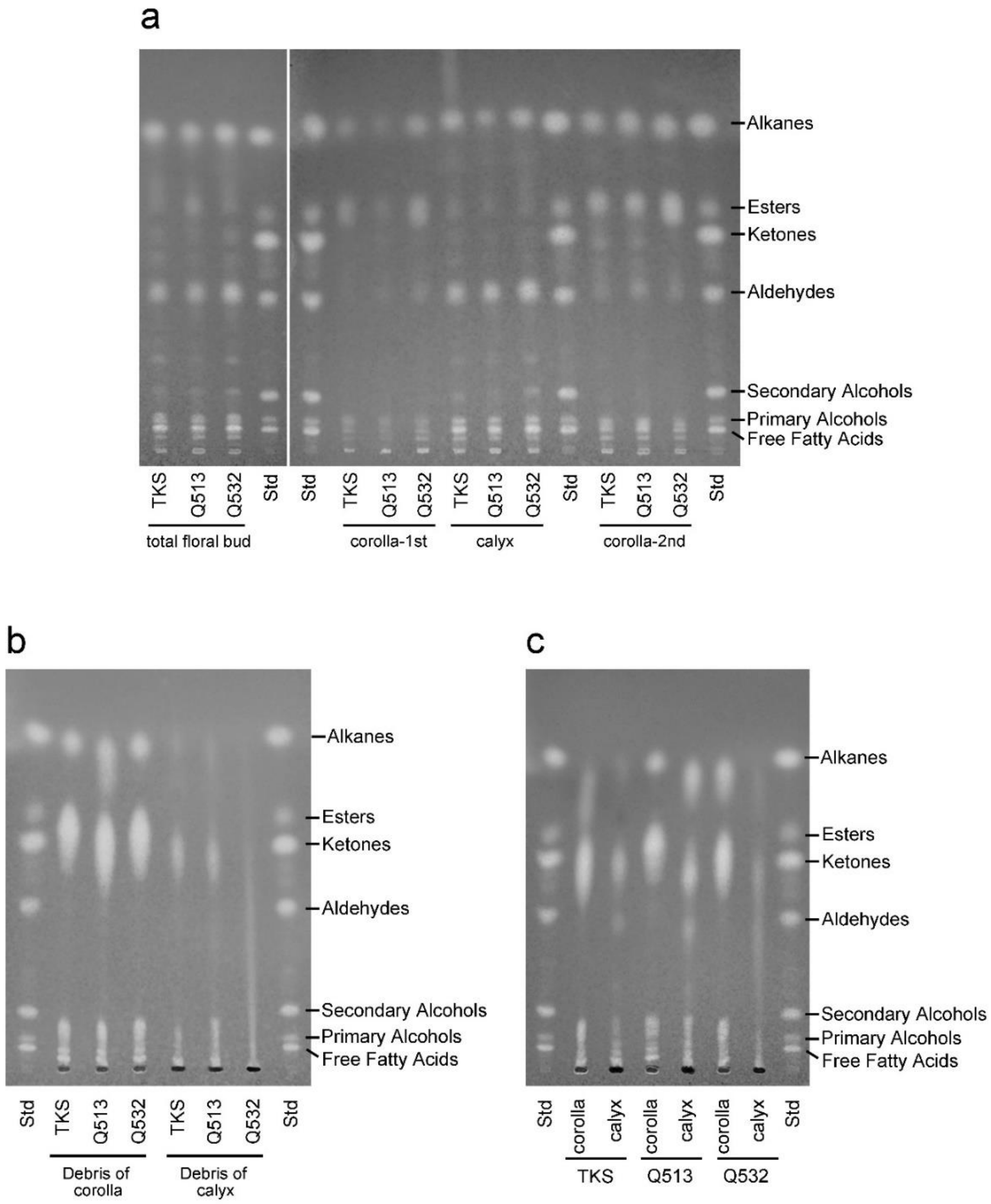

Supplementary Fig. 6. Lipid analysis by thin-layer chromatography in perianth organs in TKS and cup flower lines. **a,** Surface lipids were extracted in $CHCl_3$ 2 times for 30 seconds, and whole extractions were applied to the material in each lane. **b,** Debris after surface lipid extraction was used for further extraction, and 0.3 mg was added to each lane. **c,** Total lipids were extracted from corolla and calyx from 3 floral buds per sample.

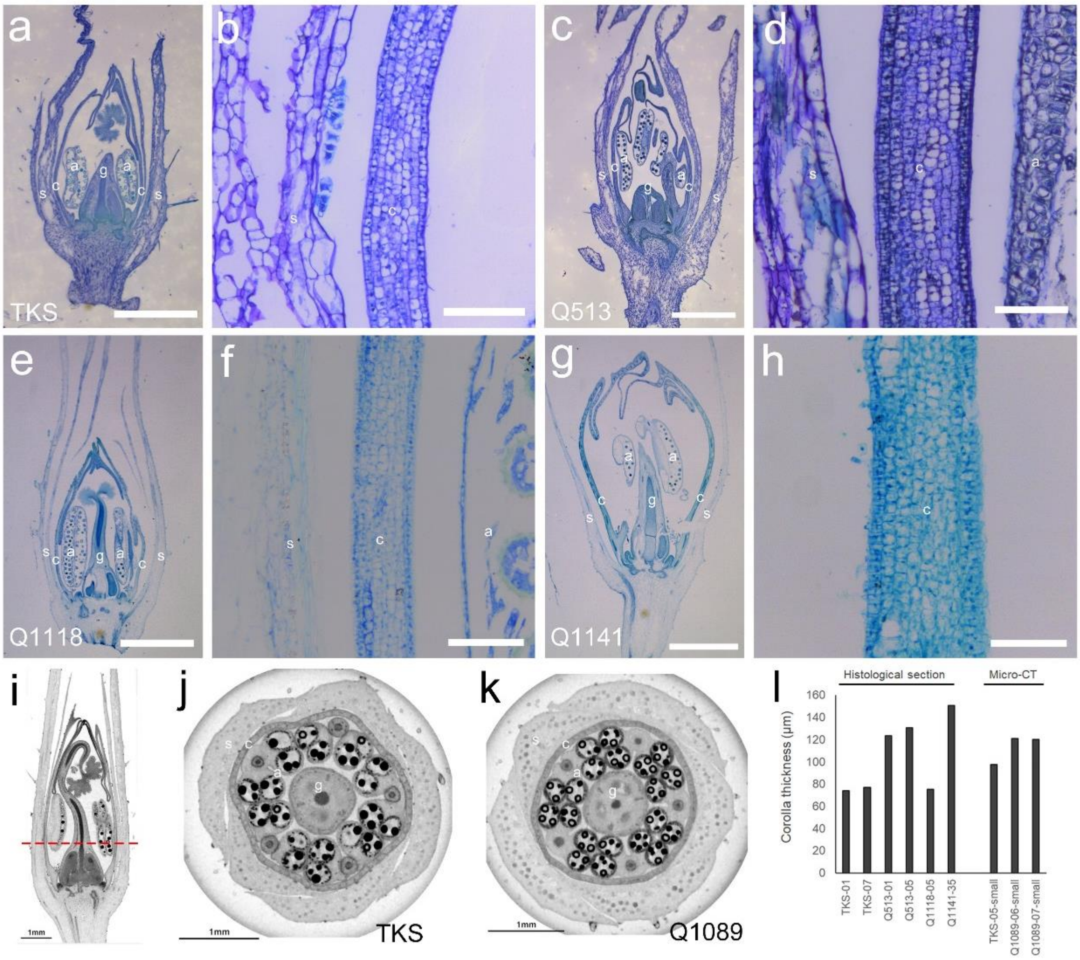

Supplementary Fig. 7. Sections of floral buds and corollas. **a-h,** Histological sections of floral buds. **a, c, e, g,** Longitudinal sections. **b, d, f, h,** High magnification of the corolla. **a and b,** TKS. **c and d,** Q513. **e and f,** Q1118. **g and h,** Q1141. **i-k,** Micro-CT images. **i,** Longitudinal section. **j and k,** Transverse sections of TKS (j) and Q1089 (k) at the plane of the red broken line in **i**. Note that there was no significant difference in corolla thickness. s: sepal, c: corolla, a: anther, g: gynoecium. **l,** Corolla thickness measured in sections. The average of a 10-point measurement is shown. Note that the corolla of the cup flower mutants is thicker than that of the wild type. Scale bars: **a, c, e, g**, 2 mm; **b, d, f, h,** 100 μm.

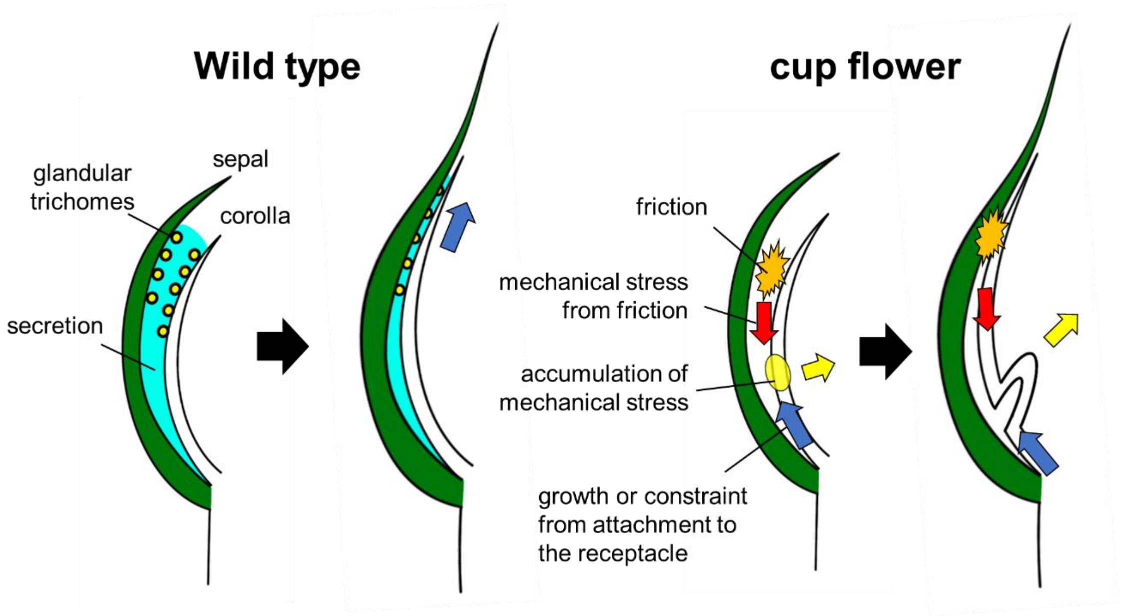

Supplementary Fig. 8. Schematic model of corolla elongation. Due to the lack of GSTs and secretion in the cup flower mutant, friction occurs between sepals and the corolla, generating mechanical stress in a downward manner. Together with stress from the constraint below due to growth or attachment to the receptacle, mechanical stress from above due to friction accumulates in the corolla and generates buckling towards the inside. Green, sepal; white, corolla; yellow circle, GST; pale blue, secretions from GSTs.

Supplementary Table 1. Lines of *I. nil* and *I. purpurea* used in this study.

**Line ID**	**Genotype[1]**	**GT type[2]**
I. nil		
Q1065 (TKS)	WT	GT
Q510	***cp*** *v1 mg pr tw*	H
Q513	***cp*** *cm1 Co ig int tw*	H
Q531	***cp*** *y1 dg dk-m int*	H
Q532	***cp-r*** *dg mg pr a3-Mr*	Fl
Q533	***cp-r*** *p pr*	Fe
Q1017	***cp*** *mg pr lt*	H
Q1089	***cp*** *y1 cm1 dy tw*	H
Q1096	***cp-r*** *y1 mg pr a3-Mr*	Fe
Q1097	***cp*** *y1 cm1 int*	H
I. purpurea		
Q1118	purpurea: pale pink splash/seed variegated	GT
Q1141	purpurea: ***cp*** seed variegated *y-m*	D

1. The genotype information was obtained from the Morning Glory Homepage (http://mg.biology.kyushu-u.ac.jp/)

2. Type of trichomes on petals. GT, glandular: H, hairy: Fl, flat: Fe, few: D, deformed.

Supplementary Table 2. Sequences of primer used for RT-PCR.

Primer name	Sequence (5' to 3')
InMYB113_F	CCTGGACGGAACAGGAAGATAATC
InMYB113_R	ACGTCATTCCGTCACCGTCGAATT
InbHLH35e_1F	ATGGAAAACATTGGAGAAAACATC
InbHLH35e_4R （InbHLH35_R）	CTAGATGCTCATTGGACTGTCTGG
InNAC029e_F	ATGGTGGCTAAAAACAGGTCGTCT
InNAC029e_R	TCACTGGAACTGGAATGCAACAGG
InTCP2e_2F	GCAAGGTGTGGATTTCCAAGGGGT
InTCP2e_R	TTAGAACCAATTATCCGCAGGAGC
InWRKY9e_F	ATGGATATCGATTTGTCCTTGAAG
InWRKY9e_R	CTAGGATGGCGGCTTATCTTTATT
InActin_F	GAAAGATTTGTATGGAAACGTCGTG
InActin_R	CGAAATCCACATCTGTTGGAAAGTA
