# Supplementary material for: Reduction in organ–organ friction is critical for corolla elongation in morning glory
Source: Commun Biol. 2021 Mar 5;4:285. doi: 10.1038/s42003-021-01814-x (PMC7935917; doi:10.1038/s42003-021-01814-x)
Supplement: Supplementary file 3 — Description of Additional Supplementary Files [file 42003_2021_1814_MOESM3_ESM.pdf]

Description of Additional Supplementary Items

File Name: Supplementary Movie 1

Description: False-color 3D volume-rendered image generated from micro-CT scans showing the floral buds of TKS (left) and the cup flower line Q1089 (right). Note that the vascular bundles in the Q1089 corolla do not break.

File Name: Supplementary Movie 2 to 4

Description: Computational modeling of corolla elongation. Movie 2, No friction (0). Movie 3, Under mild friction (0.4), the corolla folds slightly. Movie 4, Under severe friction (0.7), the corolla folds dynamically, mimicking the cup flower phenotype.

File Name: Supplementary Data 1

Description: 101 Down-regulated genes in Q532 corolla compared to TKS.

File Name: Supplementary Data 2

Description: Source data for the Fig 1g.
