# Supplementary figures and images for: Reduction in organ–organ friction is critical for corolla elongation in morning glory
Source: Commun Biol. 2021 Mar 5;4:285. doi: 10.1038/s42003-021-01814-x (PMC7935917; doi:10.1038/s42003-021-01814-x)

Supplementary Data 2. Source data for Fig. 1g

|        | TKS (50) | Q532 (176) | Q533 (119) | Q1096 (256) | Q531 (185) | Q510 (140) | Q1017 (122) | Q513 (127) | Q1097 (104) | Q1089 (67) | Q1118 (53) | Q1141 (62) |
|--------|----------|------------|------------|-------------|------------|------------|-------------|------------|-------------|------------|------------|------------|
| Cup    | 0        | 46         | 35         | 83          | 45         | 85         | 84          | 101        | 90          | 64         | 0          | 48         |
| Half   | 0        | 28         | 26         | 121         | 46         | 32         | 17          | 19         | 11          | 2          |            | 7          |
| Traced | 0        | 21         | 19         | 20          | 47         | 10         | 15          | 2          | 2           | 0          | 0          | 3          |
| Normal | 50       | 81         | 39         | 32          | 47         | 13         | 6           | 5          | 1           | 1          | 53         | 4          |
| Total  | 50       | 176        | 119        | 256         | 185        | 140        | 122         | 127        | 104         | 67         | 53         | 62         |

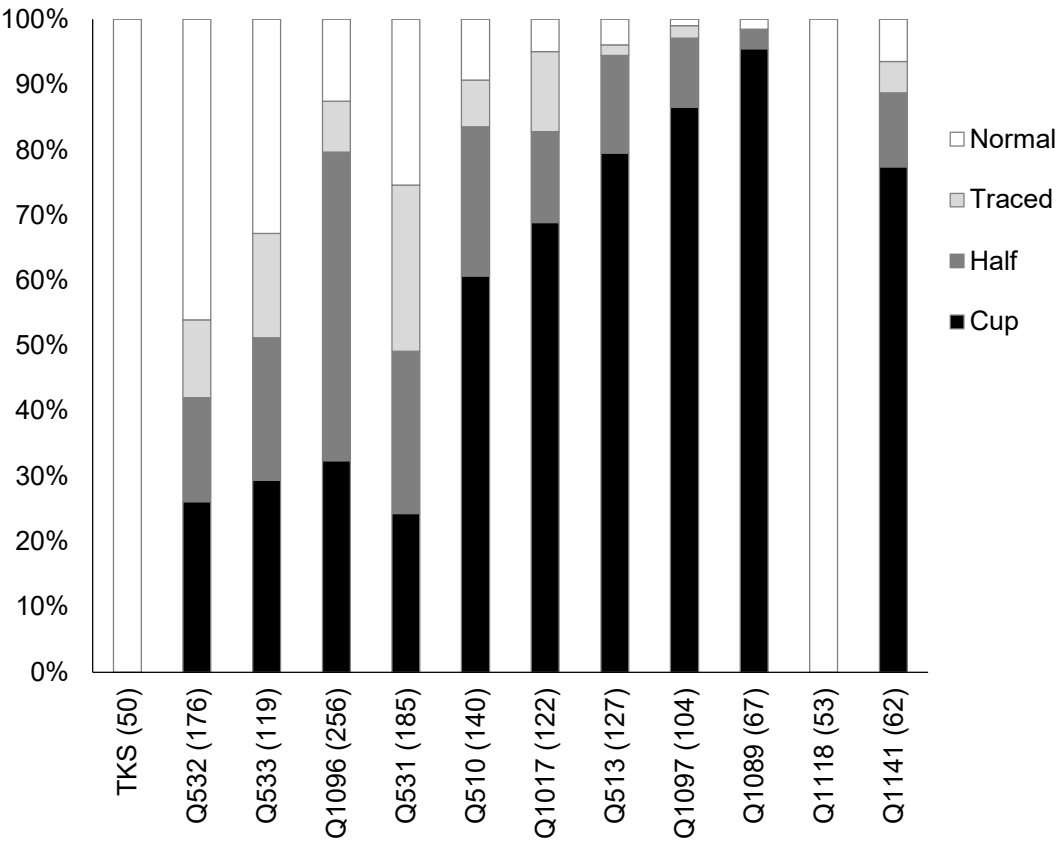

Supplement: Supplementary file 9 — Supplementary Data 2 [file 42003_2021_1814_MOESM9_ESM.pdf]
